# Tranexamic acid reduces blood cost in long-segment spinal fusion surgery: a randomized controlled study protocol: Retraction

**DOI:** 10.1097/MD.0000000000028813

**Published:** 2022-02-11

The article, “Tranexamic acid reduces blood cost in long-segment spinal fusion surgery: A randomized controlled study protocol”,^[1]^ which appeared in Volume 99, Issue 37 of *Medicine* is being retracted. This article was initially published with an incomplete table provided by the authors, and an erratum published in anticipation of the corrected version. The authors have not responded to multiple requests, and as a result, the article has been fully retracted.
